# Biophysical and Structural Studies on the Capsid Protein of the Human Immunodeficiency Virus Type 1: A New Drug Target? 

**DOI:** 10.1100/tsw.2009.52

**Published:** 2009-05-29

**Authors:** José L. Neira

**Affiliations:** ^1^ Instituto de Biología Molecular y Celular, Universidad Miguel Hernáez, 03202 Elche (Alicante), Spain; ^2^ Biocomputation and Complex Systems Physics Institute, 50009 Zaragoza, Spain

**Keywords:** protein structure, binding, CAC protein, lipids, protein-peptide interactions, protein-protein interactions, organic molecules

## Abstract

AIDS affects 30 million people worldwide and is one of the deadliest epidemics in human history. It is caused by a retrovirus, HIV, whose mature capsid (enclosing the RNA with other proteins) is formed by the assembly of several hundred copies of a protein, CA*. The C-terminal domain of such protein, CAC, is a driving force in virus assembly and the connections in the mature capsid lattice indicate that CAC joins through homodimerization of the CA hexamers. In the first part of this work, I shall review the biophysical studies carried out with the dimeric wild-type CAC protein and a mutant monomeric variant. The results open new venues for the development of drugs able to interact either with the dimeric species, hampering its assembly, or with the monomeric species, obstructing its folding. In the second part of this review, I shall describe the structures of complexes of CAC with small molecules able to weaken its dimerization. Furthermore, interactions with other proteins and lipids are also described. The whole set of results suggests that much of the surface of CAC does not accommodate binding per se, but rather binding sites in the protein are predefined, i.e., there are “hot” spots for binding in CAC (whatever be the molecule to bind). These “hot” residues involve most of the dimerization interface (an α-helix) of the CAC wild-type protein, but also polypeptide patches at the other helices.

## INTRODUCTION

HIV-1 (human immunodeficiency virus type I), the agent responsible for AIDS, belongs to the retroviral family. Understanding the life cycle of HIV-1 has fueled the development and application of diverse therapeutic strategies. Targets for intervention include the inhibition of the fusion of the virus at the surface of the CD4+ T cells, the HIV maturation, the reverse transcription of the viral RNA, and processing of the Gag polyprotein by the HIV-1 protease[1]. However, rapid emergence of drug-resistant

*Abbreviations list appears at end of paper.
©2009 with author.
Published by TheScientificWorld; www.thescientificworld.com variants of the virus has yielded therapeutic strategies partially ineffective, making AIDS a chronic illness.

As are other retroviruses, the mature infective HIV-1 is composed of several shells that facilitate the early stages of viral replication. The external surface of the virion is formed by a lipid bilayer, where transmembrane envelope proteins are embedded. The internal surface of this bilayer is also coated by a protein matrix shell composed of the so-called matrix protein, MA. Within this envelope formed by MA and the lipid bilayer, there is a conical capsid formed by the assembly of several hundred units of the so-called capsid protein (CA) surrounding a viral complex formed by the RNA genome, the nucleocapsid protein (NC), and viral enzymes. Recent insight into the structural biology of the virus particle itself[2] and the assembly mechanism of its conical capsid have made the CA protein emerge as an important new target for drug development[3]. Assembly can be considered as a good target for antivirals since it depends only on repetitive weak protein interactions, and probably disruption of a small part of these interactions is sufficient to suppress infectivity in a dominant-negative effect. In addition, the capsid plays critical roles in the early stages of viral replication and it interacts with innate immunity factors[4].

Capsid assembly occurs through the controlled polymerization of the Gag polyprotein that is transported to the plasma membrane of infected cells, where assembly of the immature particles occurs[5,6,7]. Then, budding of spherical immature noninfectious particles, and encapsulation of the genomic material and other viral proteins, takes place. Immediately after budding, HIV maturation is initiated by proteolytic cleavage of the Gag polyprotein. Cleavage by the viral protease yields MA, CA, NC, and p6 proteins, as well as two spacer proteins. This maturation process (1) induces conformational changes in the CA domain and (2) triggers a change in the morphology of the particles, which have now formed an electron-dense core and a distinctive conical capsid shape. At this stage, the viral mature capsid is formed by approximately 1500 copies of the CA protein[8]. Retroviral capsids are organized following the principles of fullerene cones, where the hexagonal CA lattice is stabilized by several interfaces. This process has a critical role in viral infectivity since the defective formation of the capsid results in noninfective particles. Furthermore, mutations in the CA protein lead to defects in viral assembly and core condensation[9,10]. *In vitro*, CA spontaneously assembles into cylindrical structures and cones resembling the viral capsid[11,12,13,14,15].

The CA protein of HIV-1 is formed by two independently folded domains separated by a flexible linker[16,17,18,19]. The CAN (residues 1–146 of the intact protein) is composed of five coiled-coil α-helices, with two additional short α-helices following an extended proline-rich loop[16,17,18]. The CAC domain (residues 147–231) is a dimer both in solution and in the crystal form[19,20]. Each CAC monomer is composed of a short $3_{10}$-helix followed by a strand and four α-helices: α-helix 1 (residues 160–172), α-helix 2 (residues 178–191), α-helix 3 (residues 195–202), and α-helix 4 (residues 209–214), that are connected by short loops or turn-like structures (Fig. 1). The dimerization interface is formed by the mutual docking of the α-helix 2 from each monomer, with the side chains of each tryptophan (Trp184) deeply buried in the dimer interface[19,20]; this helix has a kink at position Thr188. The two additional aromatic residues in each monomer, Tyr164 and Tyr169, are located in the hydrophobic core of each monomer, well away from the dimer interface (Fig. 1). Dimerization through CAC is a driving force in virus assembly[2,14,15,21,22] and recent studies of the mature capsid lattice have shown that CAC connects through homodimerization of CA hexamers, mediated by CAN, which form the mature capsid[2 and references therein; 23]. We have used the CAC dimeric interface as a model to describe the interactions governing assembly of HIV-1 and to understand, in turn, the forces behind the quaternary structure of oligomeric proteins. Moreover, on the basis of such protein engineering studies, we have started to design peptides able to interact with such an interface that could be used as lead molecules for drug design.

The following review is divided in two main sections. In the first part, I review the recent developments in the biophysical characterization of CAC, taking the protein as a model for folding and assembly. The results open new venues for the development of drugs able to interact either with the dimeric species, hampering its assembly, or alternatively with the monomeric species, obstructing its

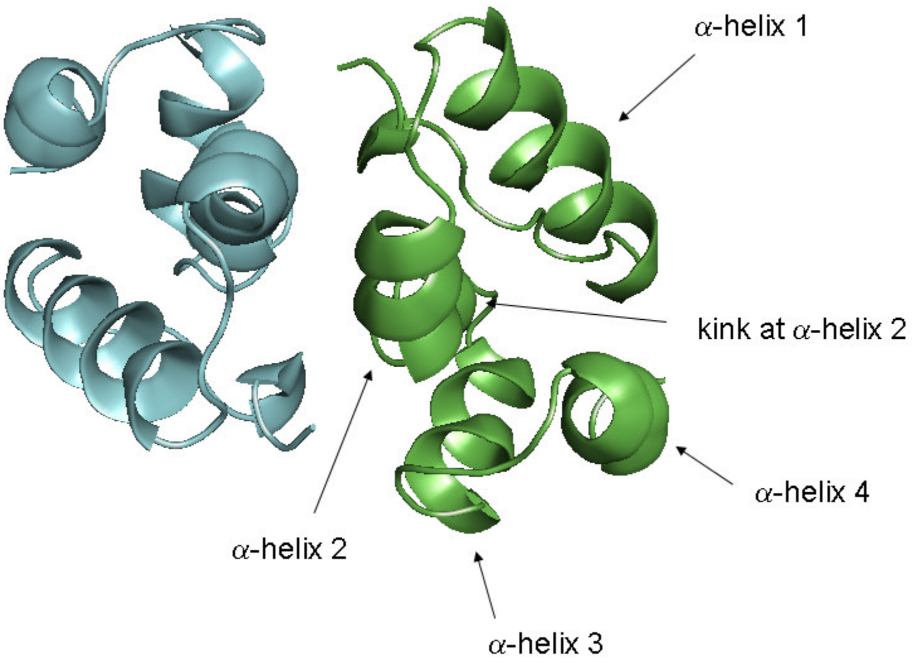

**FIGURE 1**. Structure of the CAC dimer. X-ray structure of CAC showing the dimeric structure of the domain. The monomers are depicted in different colors (blue and green); the monomer on the left is oriented with the axis of the dimerization helix perpendicular to the figure. The figure was produced with PyMOL (DeLano Scientific)[69] (PDB file accession no. 1a43). The different elements of secondary structure and the kink at the second α-helix are indicated in the right-side monomer.

folding. In the second part, I review how the native dimeric structure can be modified by the presence of mutations, small polypeptides (which can be considered assembly inhibitors), or even lipids. The whole set of results suggests that much of the surface of CAC does not accommodate binding *per se*, but rather binding sites are predefined, i.e., there are “hot” spots for binding (whatever be the molecule to which CAC binds).

## BIOPHYSICAL STUDIES OF CAC

### CAC as a Model Folding Protein: Biophysical Characterization

Some proteins carry out their biological functions as multimeric assemblies; in particular, as dimers[24]. Most of the work on protein folding in the last years has focused on small monomeric single-domain proteins[24,25,26,27]. These proteins, on titration with denaturants like urea or guanidinium chloride (or when heated), display a highly concerted transition between the denatured (D) and the native (N) state. This all-or-none process agrees with a funnel-shaped energy landscape, where the bottom of the funnel corresponds to the native state. Furthermore, this shape of the energy landscape funnel indicates that folding proceeds towards the folded state through a nonrandom process, probably involving several folding routes. This, in addition, suggests that folding occurs through the formation of local elementary structures of different stability. The formation and assembly of these elemental structures are closely related to the transition states and to the folding nucleus of the whole intact protein[25,26].

Further complications arise in multimeric proteins, where their folding involves not only intramolecular interactions (as in monomeric proteins), but also intermolecular ones. How the amino acid sequence controls subunit-subunit interactions, and which additional stability is conferred by such contacts, can be only addressed by characterizing the stability and conformational properties of model multimeric proteins. In these oligomeric macromolecules, the normal behavior is the presence of, at least, an intermediate state between the denatured and native states, which can be either monomeric or multimeric, having native-like structure in some regions[28]. For instance, in neutral solution, the dimeric HIV-1 protease folds according to a three-state mechanism ($2D \leftrightarrow 2N \leftrightarrow N_2$), populating a monomeric native-like conformation (see [29] and references therein). Then, each monomer is stable enough to fold independently through the same mechanism as a monomeric chain and, then, each one diffuses and collides to yield the native dimer, whose dissociation constant is 5.8 μM (at 4ºC)[30].

The unfolding behavior of CAC is quite different, although it also shows uncoupling of dissociation and unfolding. First, CAC unfolds differently whether temperature or a chemical agent is used as denaturant (Fig. 2). Three reversible thermal transitions are observed, depending on the technique employed (Fig. 2A). The first one is protein concentration–dependent, and observed by FTIR and NMR, consists of a broad transition occurring between 290 and 315 K; this transition must involve dimer dissociation. The second transition (with a $T_m$ ~ 325 K) is observed by ANS-binding experiments, fluorescence anisotropy, and near-UV CD; it involves partial unfolding of the monomeric species. Finally, absorbance, FTIR, far-UV CD, and NMR revealed a third transition occurring at $T_m$ ~ 333 K, which involves global unfolding of the monomeric species[31]. The first transition is also monitored by the broadening of the resonance of the indole moiety of Trp184, suggesting that its mobility and/or the conformational equilibria affecting such an aromatic group are dramatically affected on dissociation. The structural properties of the proteins populated during the second transition are similar to those observed in molten-globule species[32], and appear to resemble the features of a partially folded species observed at low pH in CAC[31]. The several independent spectroscopic techniques used allow the determination of the $\Delta C_p$ (the heat capacity change) of the thermal denaturation of monomeric CAC. This value was ~1.3 kcal $mol^{-1}$ $K^{-1}$, which is similar to those observed in other monomeric proteins of comparable size[33]; in contrast, the values of the enthalpy (74 ± 8 kcal (mol of cooperative unit)$^{-1}$) and the entropy (230 ± 30 cal $K^{-1}$ (mol of cooperative unit)$^{-1}$) of the dissociation reaction are small when compared with those of other oligomeric proteins[34 and references therein]. The exact reasons for such small value are unknown, but they could be due to (1) experimental errors due to the technique used in the determination of the thermodynamic parameters (FTIR), or (2) the fact that most of the values reported in the literature for oligomeric proteins come from protein dissociation reactions where dissociation and unfolding occur concomitantly at the same step of the folding reaction, and in CAC the final dissociated monomeric state is a well-folded monomer, which later on, in another sequential reaction, unfolds completely.

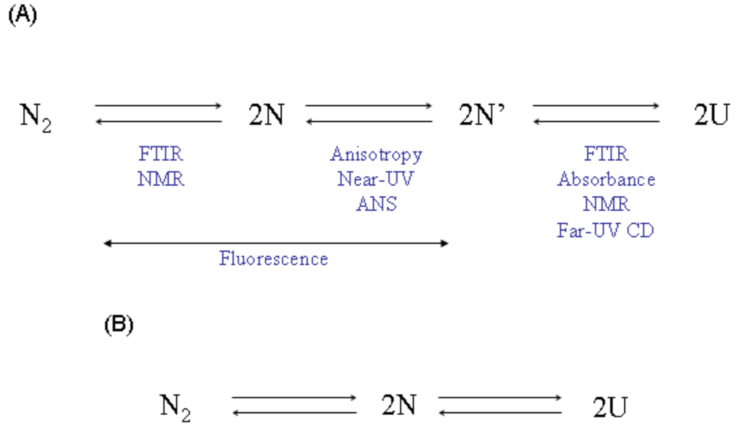

**FIGURE 2**. Equilibrium denaturation of CAC. The picture shows the different species populated through the denaturation of CAC by thermal (A) or chemical (B) denaturations, with the probes used for identification of each conformational step.

Conversely, when equilibrium unfolding of CAC is followed by chemical denaturation, only two transitions are observed, consistent with a three-state model ($N_2 \leftrightarrow 2I \leftrightarrow 2D$)[35] (Fig. 2B). The dimer ($N_2$) dissociates into a monomeric intermediate (I) of very low conformational stability. This intermediate fully preserves the native secondary structure, but it has lost part of the tertiary (intramonomer) interactions found in the dimer. In a second transition, the intermediate cooperatively unfolds into denatured monomer (D). Two different techniques have been used when describing the chemical-denaturation unfolding of CAC: fluorescence and CD. The transition probed by fluorescence is protein concentration–dependent, with solvent exposure of Trp184, located at the dimeric interface. Therefore, this transition involves dissociation of the dimer into a monomeric form. However, there is a large discrepancy between the $K_U$ (the equilibrium constant obtained from the chemical-denaturation curves for the equilibrium: $N_2 \leftrightarrow 2I$, with a $\Delta G$ = 12.1 kcal mol$^{-1}$, which yields a $K_U$ = 1.2 nM) as measured by fluorescence[35] and the $K_D$ (10 μM) determined by equilibrium centrifugation[19]; this discrepancy indicates that the transition probed by fluorescence cannot correspond to the rigid dissociation into fully native monomers and, thus, additional conformational rearrangements must occur. On the other hand, the transition observed by far-UV CD is protein concentration–independent and, thus, must be related to unfolding of a monomeric intermediate. This transition shows a high cooperativity, and the corresponding *m*-value is not very different from the values obtained for some monomeric proteins of similar size whose unfolding equilibrium involves only the fully native and fully denaturated forms (as it occurs with the $\Delta C_p$ value determined from the thermal denaturations)[27,33]. Then, how can both equilibrium unfolding reactions (chemical and heat) be reconciled? Based on the above data, one of the simplest explanations suggests that chemical denaturation experiments followed by fluorescence spectroscopy must be reporting the two first steps of the thermal unfolding reaction, i.e., dissociation and the small rearrangement of the native-like monomer[35] (Fig. 2B).

In conclusion, as it occurs in HIV-1 protease and what could look surprising for a small protein, CAC unfolds following a three (or four)-state mechanism. We hypothesize that the intermediate species is a consequence of the dimeric nature of the protein. Thus we can raise the question, is it possible to find the conditions where some of those monomeric species could be highly populated? If so, could we study them at equilibrium? We have observed that at medium-to-high temperatures, the Trp184 of the monomeric species changes its environment and/or its conformational equilibria (as shown by the broadness of the signal in the NMR spectrum). Therefore, the simplest way to use such equilibrium to characterize the presence of possible monomeric species is to study the species present at 330 K. However, the duration of the NMR experiments and the partially unfolded character of the species at these temperatures have led to precipitation in all assays (unpublished results). Thus, we have used another alternative approach: designing a mutant protein that could stabilize the monomeric species at equilibrium.

## The Monomeric Species of CAC: Structure and Flexibility

If we want to stabilize the monomeric species, we must understand first, as much as possible, the whole thermodynamics governing the quaternary, tertiary, and secondary structures of CAC. To that end, we carried out a description of the stability of the wild-type protein[31,35]. In addition, del Alamo et al. performed an alanine scanning to obtain an estimation of the contribution of each side chain to the helical dimeric interface[36]. The amino acid side chains per monomer that participate in intersubunit contacts at the CA dimeric interface were truncated to alanine to analyze their individual energetic contribution to protein association and stability[36]. Different biophysical techniques were used to estimate (1) the affinity constant for each mutant by gel filtration (monitoring the quaternary structure), (2) the protein dissociation and the conformational rearrangements by fluorescence spectroscopy (monitoring tertiary and secondary structures), and (3) unfolding of the native-like monomer by far-UV CD (monitoring the secondary structure). The results show that about half of the side chains in the contact epitope (the α-helix 2) are important in the energetic epitope, as their truncations essentially prevent dimerization. However, the global dimerization affinity is low due to the presence of interfacial side chains whose individual truncations improve affinity by two- to 20-fold. Thus, there are several classes of side chains in the wildtype dimeric CAC, those which stabilize the dimer and those directly destabilizing it. Furthermore, there is an interplay between quaternary and secondary structure within the monomer since many side chains at the interface are also energetically important for the folding of the monomeric intermediate and for its conformational rearrangement during dimerization. Therefore, it is not trivial to separate contributions from quaternary, tertiary and secondary structures.

The alanine scanning study also suggests that the most destabilizing mutation was Trp184 to Ala (CACW184A) and that the second-most destabilizing mutation was Met185 to Ala (CACM185A). Both mutants were previously found to be essentially monomeric even at high concentrations[19]; we have tested that the CACW184A mutant is monomeric up to millimolar concentrations and, then, amenable for NMR structural studies. The structure of this mutant is similar to that of the subunits in the dimeric, nonmutated CAC except the segment corresponding to the second helix, which is highly dynamic (Fig. 3A), sampling different conformations (and among them, the helical native-like structure)[37]. At the end of this second helical region, the polypeptide chain is bent to bury several hydrophobic residues and, as a consequence, the last two helices are rotated 90° when compared to their position in dimeric CAC. Therefore, we have proposed that the monomeric structure of the CACW184A must be similar to that of the monomeric species N' in Fig. 2A, and the bending in the loop, following the second helix, must explain the rearrangement detected by fluorescence measurements and that occurs during chemical denaturations concomitantly to dimer dissociation[37].

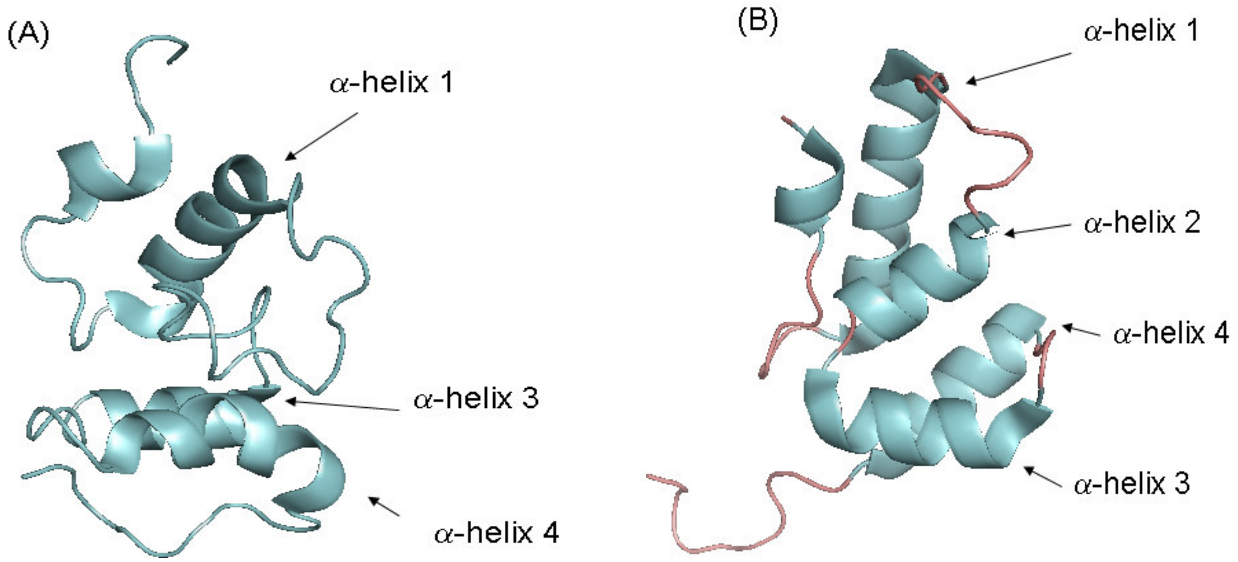

**FIGURE 3**. Structures of monomeric CAC. The structure of the CACW184A monomeric mutant[37] (PDB file accession no. 2jo0) (A) and that of the double monomeric mutant CACW184AM185A[38] (PDB file accession no. 2jyl). The figures were produced with PyMOL (DeLano Scientific)[69]. The helices are named after the elements of structure in the wild-type CAC in Fig. 1.

The NMR structure of the double mutant CACW184AM185A has also been reported[38]. The double mutant is also monomeric, with a shortened α-helix 2 (when compared to the wild-type protein[19]), lacking the kink at Thr188 seen in the X-ray structure of wild-type CAC dimer. Interestingly enough, there is no evidence of the bending of the loop following α-helix 2, and then the last two helices (Fig. 3B) are not rotated when compared to their position in the wild-type protein. Then, how can these differences in the structure of the isolated monomer be rationalized? The most plausible explanation is the different pH values used in the two experimental sets. Whereas the structure of CACW184A has been solved at pH 7, that of the double mutant is solved at acidic pH. Furthermore, Krishna and coworkers describe that as the pH is raised, the resonances of residues belonging to the native-like, well-formed α-helix 2 in

CACW184AM185A disappear[38]. These data suggest that the structure of the monomeric species is highly pH dependent, probably due to the two glutamic acid residues present in the dimerization helix. Interestingly enough, the nonmutated CAC forms a similar dimer at low pH as that observed at physiological pH[19], but the dimeric interface is more hydrophilic, with a different interdomain angle[2]. These tertiary pH-dependent conformational changes are not unusual in proteins and, for instance, they have been observed in the quaternary structure of the C-terminal domain of the influenza M2 protein, where a lysine residue is able to change the arrangement of the monomers within the tetramer[39], or, in the dynein light chain LC8, where mutation of a histidine residue causes monomerization[40].

Although the structure of α-helix 2 is flickering in monomeric CACW184A[37], we conclude that it is present in the isolated monomeric species of CAC, as suggested by the structure of CACW184AM185A[38], and by the FTIR and far-UV CD results. These findings strengthen the view that rather than providing a general and unspecific binding surface, CAC contains a small number of binding hotspots that can be considered actual binding sites. If this holds for other proteins (as computational studies seem to pinpoint[41] and more experimental studies support[40,42,43]), then some areas of the protein surface could be described *a priori* as “hot” binding sites. This hypothesis seems to hold for CAC, and it is also reinforced by NMR dynamic studies of CACW184A in the timescale from nsec to psec[42]. Residues with slow conformational exchange (in the msec time regime, i.e., in equilibria among different conformers), and then exploring several highly flexible structures, are (1) those belonging to the second helix or close to it, and (2) those suffering any type of conformational rearrangement as a consequence of the flickering helical structure[42].

## CAC AND THEIR INTERACTIONS WITH OTHER MOLECULES

### Dimeric CAC as a Drug-Target Protein: Dimerization Inhibition Studies

If we want to use CAC as a possible drug target against HIV-1, the biophysical studies above suggest that novel inhibition approaches should aim to destabilize either the dimeric or the monomeric form. In principle, destabilization of the dimeric species of CA homodimerization and, then, HIV capsid assembly, could be carried out by using isolated CAC as a competitive inhibitor[44,45]. Alternatively, virion assembly can be hampered by the use of small peptides or organic molecules that bind to the single monomers of CAC, thus occupying the dimerization interface and then obstructing binding to the other monomer. Also, assembly inhibition can be directed against the whole Gag processing, as it has been reported for a small organic molecule[46,47]. However, in this review, I shall focus on the design of inhibitors involving, in one way or another, CAC. In this sense, different approaches have led to three different small-molecule designs:

#### *Small Organic Molecules*

The first breakthrough in identifying small-molecule inhibitors of CAC assembly was reported by Summers’ group[48]; in fact, the molecules were not designed specifically against CAC, but rather against the whole CA. The first molecule, CAP-1, N-(3-chloro-4-methylphenyl)-N'-{2-[({5-[(dimethylamino)-methyl]-2-furyl}-methyl)-sulfanyl]ethyl}-urea) (Scheme 1), has dose-dependent inhibition in viral infectivity assays, and its affinity for CAN is in the range of 800 μM. Structural studies show that CAP-1 binds in a pocket formed at the point where helices 1, 2, 4, and 7 of the CAN interact[49]. Helices 4 and 7 of CAN form the interface required to create a stable hexagonal CA lattice of the virion capsid[2,23,50], and these helices pack against α-helices 1, 2 (the dimerization helix), and 4 of the CAC domain. Therefore, CAP-1 does not inhibit capsid assembly through direct binding to CAC, but rather because it hampers the proper docking of both domains of CA. The other small molecule designed by Summers’ group, CAP-2, was cytotoxic[48].

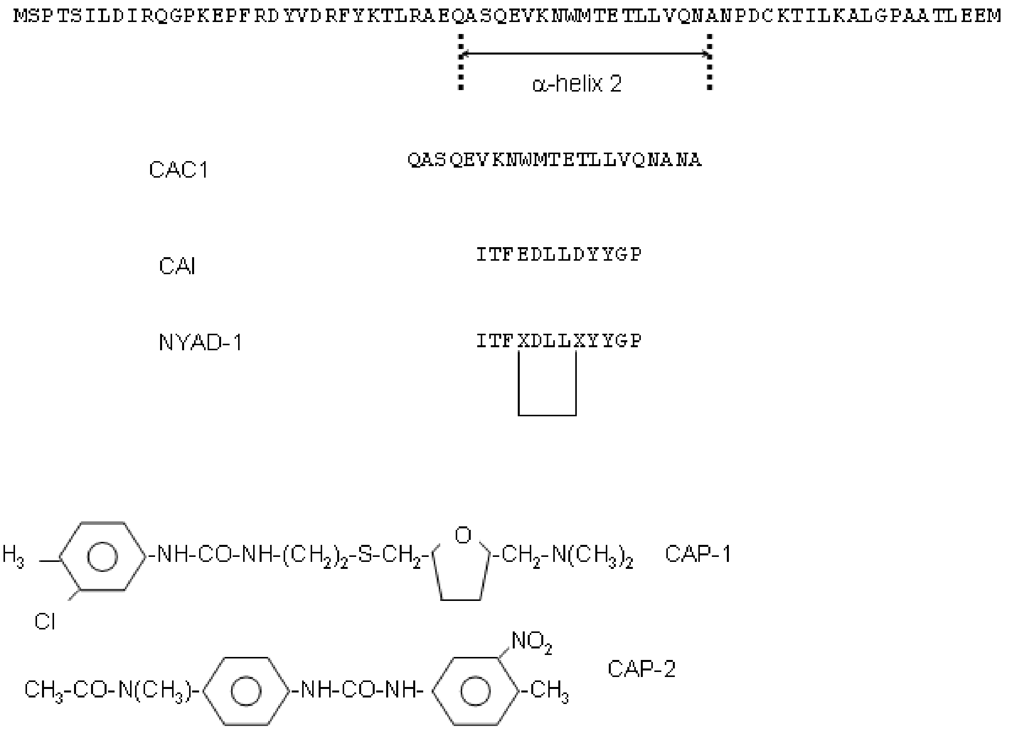

**SCHEME 1.** The sequence of the intact CAC is shown at the top. The second one is the sequence of CAC1[51], the third is that of CAI[52], and the fourth, with the hydrocarbon staple between two residues, is that of NYAD-1[55]; the X indicates the target amino acids for the hydrocarbon stapling, which is indicated by the lines. All the peptides were amidated and acetylated. The last two lines show the structures of CAP-1 and CAP-2[48].

## A Peptide Mimicking the Dimerization Interface

The first peptide aimed directly to inhibit the dimerization of CAC was designed by our group and it takes as a model the design of the dimerization helix of the protein[51] (Scheme 1). Thus, we reasoned that if we wanted to inhibit dimerization, the simplest lead compound should be a peptide comprising those most important residues in assembly, which the alanine scanning had previously identified[36]. The designed peptide, CAC1, forms a complex with CAC. Thermal denaturations followed by far-UV CD, 1D-NMR spectroscopy, and size-exclusion chromatography provided evidence of the interaction between CAC1 and CAC. The apparent dissociation constant of the heterocomplex formed by CAC and CAC1 was determined by fluorescence spectroscopy (using an anthraniloyl-labeled peptide, which has the same structural and self-associating properties as the unlabelled peptide), affinity chromatography, and isothermal titration calorimetry; the three techniques yielded similar values for the apparent dissociation constant; in the order of ~50 μM. This apparent $K_D$ was only five times higher than the dissociation constant of both CAC and CA (10 μM)[19]. However, the main problem we faced with CAC1 was its high tendency to self-associate with an apparent disssociation constant (15 μM) similar to that observed for dimeric CAC. These data suggest that many important interactions for CAC recognition are contained within α-helix 2, in agreement with all the structural and thermodynamic background[36], i.e., the CAC1 peptide mimics most of the interactions that form the dimerization interface of CAC. The difference between the affinity values obtained for the peptide-protein heterodimer and the protein homodimer might be due to entropic considerations. In fact, there is a large conformational entropy penalty paid on binding of CAC1 to CAC, mainly because of the larger flexibility of CAC1 alone compared with the CAC monomer. We do not yet know, however, whether the heterocomplex is formed by only *one* single species of each macromolecule (i.e., *one* molecule of CAC1 and *one* of CAC), neither do we know the

CAC1 binding site on CAC. Experiments are underway in our laboratory using $^{15}$N-HSQC spectra to monitor the binding regions. And finally, we do not know if, on binding, the peptide adopts a native-like conformation and, then, whether maintaining the most critical residues for assembly (i.e., Trp184 and Met185), the tendency to adopt an α-helical structure improves the affinity. We have started to design peptides that decrease the self-associating tendencies of CAC1, while improving, at the same time, their association towards CAC and their helical propensity (in collaboration with Claudio Cavasotto, Houston, Texas).

## A Phage-Display Peptide and Its Variants

A third approach also involves the use of peptides that target CAC. A 12-mer peptide, CAI, was identified by Kräusslich and coworkers using phage-display techniques[52]. CAI is the first reported peptide able to disrupt the assembly of both mature- and immature-like particles *in vitro*, but due to its lack of permeability, it cannot inhibit HIV-1 in cell culture. Comparison of the sequences of CAI and CAC1 (Scheme 1) did not show any similarity; further, the sequence of CAI does not contain Trp or Met at the key positions governing the formation of the quaternary structure of wild-type CAC. Thus, in the absence of a structure of the complex between CAC1 and dimeric CAC, probably these sequence differences indicate that the mechanism of binding to CAC is quite different in both peptides.

The CAI peptide adopts an α-helical conformation on binding to CAC, as shown by the X-ray three-dimensional structure of the CAI-CAC complex[53], and it binds to a groove created by α-helices 1, 2 (the dimerization helix), and 4 of CAC; interestingly, this latter region of CAC is close to the CAP-1 binding site described above. Kräusslich and colleagues recently showed that residues Tyr169, Asn183, Glu187, and Leu211, all of which form the binding pocket site of CAI, are necessary for quaternary structure integrity of CAC[54]; all the alanine mutants at those positions are competent for immature-like assembly *in vitro* and budding of immature-like particles. Their results pinpoint an indirect (allosteric) effect of CAI binding on the assembly of the immature lattice, but a direct binding effect in the conserved CAC binding pocket during mature assembly. Interestingly enough, the mutations at Tyr169 and Leu211 do not yield mature-like particles and raise noninfectious virions with nonregular mature cores. The X-ray three-dimensional structures of the mutants at these latter positions are different to those of wild-type, and those of the assembly-competent mutants Glu187A and Asn183A; the conformations of the two assembly-incompetent mutants (Tyr169 and Leu211) in the absence of CAI are the same as the structures of any mutant when complexed to the peptide[54]. Interestingly enough, Glu187 (together with Ser178, Glu180 and Gln192) is one of the residues that decreases the association constant of CAC[36]. We speculate that since these interfacial residues, which negatively affect affinity, appear highly conserved in HIV, they may be involved in other interactions needed for completion of the HIV life cycle.

With this structural information on CAI at hand, Debnath's group used a structure-based rational design approach to stabilize the α-helical structure of CAI and to convert it into a cell-penetrating peptide[55]. The peptide (NYAD-1) contains a bridge between two residues separated four residues away; this bridge (the so-called "hydrocarbon stapling") is supposed to restrict the conformational freedom of CAI (Scheme 1). The NYAD-1 is able to penetrate cells, and disrupts the assembly of both immature- and mature-like virus particles in cell-free and cell-based systems *in vitro*. Furthermore, it shows an enhanced helicity, which seems to suggest that the higher the helicity, the higher the inhibition. The affinity of NAYD-1 for CAC is the largest reported to date, with values close to 1 μM. The authors have carried out a SAR-NMR analysis with wild-type dimeric CAC, and they have shown that the binding site of NAYD-1 to CAC encompasses residues Phe169 to Val191, destabilizing, rather than dissociating, the nonmutated dimeric CAC. These residues involve the α-helix 2 and the nearby sites to the dimerization interface. Further, the authors solved the structure of the monomeric double mutant CACW184AM185A in complex with NYAD-1, and they show that α-helix 2 is fully formed with the kink present at the Thr188[56], in contrast to what happens with the structure of the isolated monomeric double mutant[38]; the peptide binds to a hydrophobic pocket formed by residues of the four helices. When comparison with the X-ray structures of the complexes between dimeric CAC and the original CAI peptide (that from Kräusslich's group) is carried out, the differences are mostly observed in the movement of α-helix 2, which is pushed away much further from its original wild-type position in Krausslich´s design (6 Å) than in Debnath's (3 Å).

So, what have we learned from these studies? It seems that even for closely designed peptides (such as CAI and NYAD-1), there are subtle, but key, structural differences that account for the different inhibition properties exhibited *in vitro* and *in vivo*. It also seems that there is not a single way to disrupt (or better, destabilize) the dimeric CAC. And finally, we have learned that assembly of the virion capsid as a whole must not necessarily be hampered by inhibition of the dimerization of CAC alone, but rather targeting the whole CA protein and regions close to the CAC domain. To sum up, although some of these designs can be considered promising lead compounds, further structural work is necessary in order to understand in depth the whole binding mechanism of these peptides and their variants.

## Monomeric CAC as a Drug-Target Protein: Inhibition of Folding

Folding of CAC involves not only the dimerization, but also folding of a single polypeptide chain (Fig. 2). Therefore, if we are able to hamper the folding of the single chain of CAC, we will also be imparing the dimerization step. How can the folding of a single chain be obstructed? In that field, we can take advantage of the 40 years of extensive studies of folding of single polypeptide chains[25,27]. Interrupting a folding reaction can be achieved by making the polypeptide chain interact with peptides whose sequences have an intrinsic propensity to form local structures (either native or non-native). As the concentration of the peptide with the local structural tendency increases, the protein may start nucleating by the docking of the protein polypeptide chain with that of the peptide; thus, the approach requires a large concentration of the peptide (to shift the association equilibrium, following Le Chatelier's principle) since intermolecular interactions have to compete with the more stable intramolecular interactions. This association leads to nonproductive folding (i.e., a complex between the peptide and the protein) and then to a nonactive protein. This folding inhibition approach is new and it is being applied to the design of inhibitors of dimeric HIV-1 protease[29].

We have started to design peptides that comprise separately the four α-helices of CAC and the major histocompatibility region (MHR). It seems obvious that peptides that could have an intrinsic tendency to form local structure are the well-folded regions in the three-dimensional structure of CAC. We have tested so far α-helix 2, which is the dimerization helix and is found to bind to either monomeric or dimeric CAC (see above), but with a high tendency to self-associate. We are testing separately the other three helices, which are also highly hydrophobic. The MHR is a 20-residues-long region close to the N-terminus of CAC, which is highly conserved among retroviruses, and which exact function is not known. This region is hydrogen bonded to α-helix 2 (the dimerization helix)[19] and the MHR residues are involved in the association process, affecting the stability of the CAC monomer[36]. Therefore, given the large number of interactions involved, it is tempting to suggest that MHR could be directing the early steps of folding of a single CAC chain and, thus, it could be used as an inhibitor of the folding of monomeric CAC.

Even if the use of this new methodology is successful, there are still some caveats related with such an approach, such as:

1. How specific are the peptides designed with respect to other homologous proteins?
2. Is the peptide design robust enough to resist mutations? That is, if the protein mutates, are the peptides designed (against the wild-type protein) also able to interact with the mutant protein?

I do not know the answers to these questions yet, but by doing experiments with different model proteins, we should eventually fulfill the clinical potential interest of this approach.

## CAC-Macromolecular Interactions

In the above paragraphs, I discussed the possibility of inhibiting dimerization and folding of CAC by the use of other macromolecules, and I pinpointed that the domain is highly flexible. Then, is this flexibility intrinsic to the protein to allow virion assembly and to bind other proteins or macromolecules? If so, which are those other macromolecules? In the following, I shall describe the studies to date involving CAC-macromolecular interactions and how these studies can shed some light over the quaternary structure of the protein.

Recently, Collins and coworkers[57] reported the crystal structure of a domain-swapped dimer of Δ177-CAC mutant (i.e., the mutant where the Thr177 has been deleted), which resembles the nonmutated CAC dimer except that the α-helix 1 from each of the monomers swap positions. This creates a different dimeric interface for the CAC dimer (Fig. 4). The HIV-1 CAC is a structural homologue of the mammalian SCAN dimer, which was shown to exhibit a domain-swapped dimer structure[58]. The new dimeric interface of CAC involves the MHR region, thereby providing an explanation for the remarkable conservation of this region among the retroviral capsid proteins (probably underlining the importance of MHR in the folding of a single chain of CAC and then its possible use as an inhibitor of its folding). The authors proposed that the kink present in the α-helix-2 of the monomeric species of CAC[38,56] and other retroviral CA proteins is the driving force for the formation of a possible domain-swapped dimer only appearing during Gag assembly. Interestingly, residues Trp184 and Met185 appear to be involved only in a peripheral manner in the dimeric interface of the domain-swapped dimer, thereby suggesting that their mutations may not have such importance on the dimeric interface as they have in the nonmutated CAC. The implications of such discoveries within this new CAC dimeric species are not known yet.

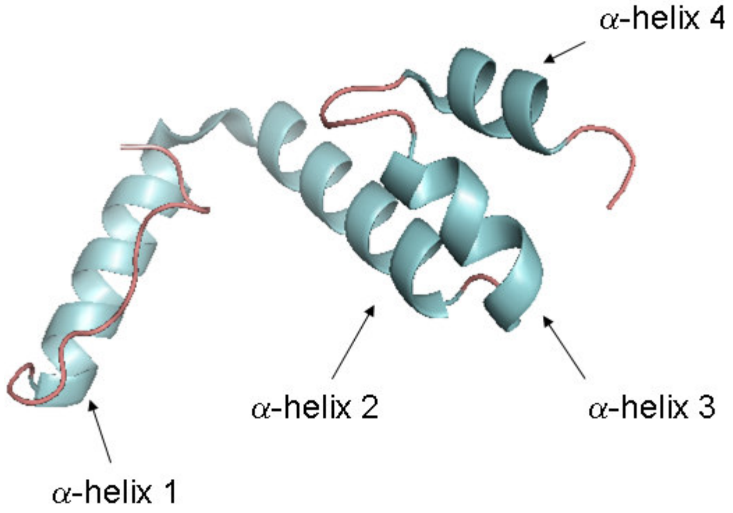

**FIGURE 4**. Structure of the swapped domain of the mutant Δ177-CAC. The figure was produced with PyMOL (DeLano Scientific)[69] (PDB file accession no. 2ont). The helices are named after the elements of structure in the wild-type CAC in Fig. 1.

A large number of the CA molecules released after Gag cleavage during virus maturation are not involved in capsid formation, and their location and function are still unknown[59,60]. On the other hand, it is not well established whether after proteolysis of the Gag protein, the MA protein, which mediates membrane binding and insertion of envelope glycoproteins into the virions[61,62], is the unique protein bound to membranes or there are other lipid-binding proteins. Thus, a complete understanding of the lipid-binding processes requires the study of the several Gag proteolytically produced proteins, and their domains, in the presence of lipids. To that end, we have shown that CAC is able to bind lipid vesicles *in vitro* in a peripheral fashion[63]. Briefly, our results show that CAC interacts strongly with the anionic lipids phosphatidic acid (PA) and phosphatidylserine (PS), whereas it displays a lower affinity for lipids without a net charge, such as phosphatidylcholine (PC), cholesterol (Cho), and sphingomyelin (SM). Interestingly enough, theoretical studies using the algorithm developed by Wimley and White[64] and von Heijne and coworkers[65] predict three different regions that are putative lipid-binding sites. The first region, Y(164)VDRFYKTL(172), is located in α-helix 1 and belongs to the MHR (Fig. 5). The theoretical prediction at this region is supported by other experimental studies on the ability of Gag protein to bind acidic phospholipid vesicles: mutation of Arg167 or the deletion of the entire MHR reduced the membrane affinity when compared with that of the wild-type Gag[66]. The second region, N(183)WMTETLLV(191), is part of α-helix 2, where the kink occurs (Thr188); and the third region, L(202)KALGPGA(209), comprises the second half of α-helix 3 and some interhelical residues. A fourth region, A(217)CQGVGG(223), also showed a tendency to interact with the membrane interface, but it produced nonsignificant scores. These four regions are closely clustered in the three-dimensional structure of the protein (Fig. 4). Most interestingly, all those regions contain residues or are close to amino acids that are critical for maintaining the quaternary structure of CAC: Trp184 and Met185 (in the second region)[36], Tyr169 (in the second one)[54], and Leu211 (close to the third region)[54]; further, α-helix 2, which the different studies described above have shown is highly flexible, and the MHR, which is involved in swapping dimerization[57], are among the predicted lipid-binding regions. Finally, the hydrophobic contacts of CAI (or NYAD-1) with wild-type CAC[53,56] or its mutants[54], namely, Val165, Phe168, Tyr169, Leu172, Lys182, Asn183, Thr186, Thr210, Leu211, and Met215, are close or involved in the above-predicted regions. Thus, we conclude that the same regions are able to interact with lipids, peptides, and other CAC molecules. However, not only is CAC able to interact with lipids, but also the whole protein[67]; we have observed that CA displays a high affinity for anionic lipids and that these are able to sequester a large population of the total amount of protein resulting in the partial inhibition of its assembly *in vitro*. We do not know at the moment whether those interactions are mediated by the contacts observed in the CAC, or alternatively CAN is also able to interact with lipids.

Finally, it is interesting to note that not only α-helix 2 interacts with other molecules, but also other helical regions of CAC do so. For instance, human $tRNA^{Lys3}$ is used as the primer for HIV-1 reverse transcription[68]. Packaging of $tRNA^{Lys3}$ into virions requires the presence, among other proteins, of the host cell factor lysyl-tRNA synthetase. This latter protein interacts with the C-terminal region of the α-helix 4 of CAC; the SAR-NMR experiments show that the chemical shifts deviations were more important for Thr210, His226, Arg229, Lys227, and Ala228, and that this intermolecular interaction was much stronger (300 nM) than that of the dimeric wild-type CAC (10 μM).

## CONCLUSIONS

From a wide point of view, the above studies suggest that the binding ability of CAC is mainly a feature of the monomer and, thus, there are some locations in the single chain of CAC that are “hot” spots for interactions. However, the “hot” binding site is not exclusively restricted to α-helix 2, but rather it appears to be modular and distributed along other regions of the protein. This modular architecture of the binding site will probably allow CAC to generate binding affinity and specificity independently within the interface formed by the several interacting polypeptide patches; this facilitates a broad range of affinities, by selecting the proper cluster of binding residues. Further, the above studies reveal that the molecular environment around the dimerization helix of CAC can be altered on binding to other molecules (lipids, peptides, or other regions of the Gag protein). It seems that the plasticity shown by the CAC domain (when faced to different molecular environments) relies mainly, although is not centered, on the second helical region (or even around Thr188, where the kink is present[57]), and that the changes in

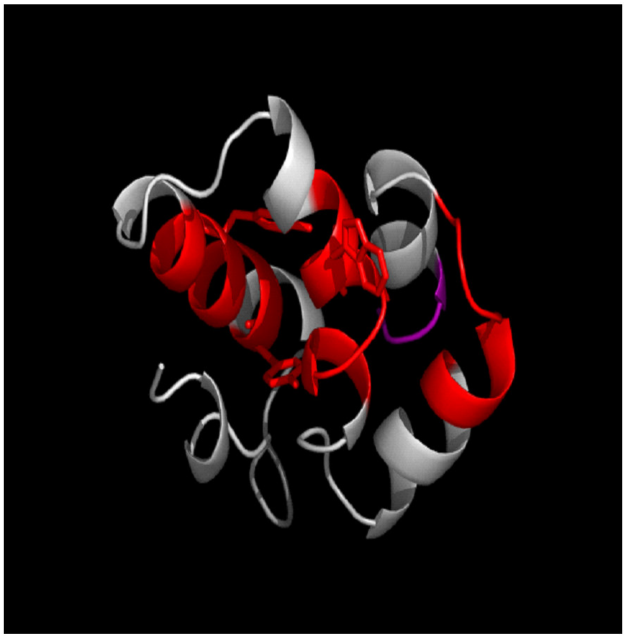

**FIGURE 5.** The interactions of CAC with lipids. Tyrosine (two) and tryptophan (one) residues are shown in sticks. The regions with predicted membrane interface affinity are depicted in red, including the region A(217)CQGVGG(223), which is in purple (the last residues V(221)GG(223) are not present in the structure). The figure was created from the PDB file 1a43 with PyMOL (DeLano Scientific)[69].

this region trigger the changes in other nearby regions. Taken together, these findings suggest that the multiple roles of CA during assembly of HIV may be mediated by its rather unique ability to adopt different conformations while preserving a similar scaffold in response to different molecular environments and/or the presence of biomolecules.

## ACKNOWLEDGMENTS

We deeply thank the two anonymous reviewers for helpful suggestions and corrections. This work was supported by grants from the Spanish Ministerio de Sanidad y Consumo (FIS 01/0004-02), Ministerio Ciencia e Innovación (SAF2008-05742-C02-01; CSD2008-00005), and the private Fundación para la Investigación y Prevención del SIDA en España (FIPSE) (Exp: 36557/06). We want to thank Dr. Nuno C. Santos for handling this manuscript as an editor. We thank Drs. Mauricio G. Mateu and Francisco N. Barrera for critical reading of the manuscript and for collaboration during all these years. We deeply thank May García, María del Carmen Fuster, Javier Casanova, and Olga Ruiz de los Paños for excellent technical assistance.

## ABBREVIATIONS

CA, capsid protein of HIV-1 (p24); CAC, C-terminal domain of CA, comprising residues 147–231 of the intact CA protein; CAN, N-terminal domain of CA, comprising residues 1–146 of the intact CA protein; CACW184A, mutant of CAC with Ala instead of Trp at position 184 of CA; CACW184AM185A, the double mutant of CAC with Ala residues at the positions 184 and 185; CAI, a phage display peptide designed by Kräusslich's group; CAC1, a peptide comprising residues Gln175 to Ala194 of the intact

CAC; CAP-1, N-(3-chloro-4-methylphenyl)-N'-{2-[({5-[(dimethylamino)-methyl]-2-furyl}-methyl)-sulfanyl] ethyl}-urea); CD, circular dichroism; FTIR, Fourier transform infrared spectroscopy; Gag, the structural polyprotein of retroviruses; HSQC, heteronuclear single quantum coherence; MHR, major homology region; MA, matrix protein; NMR, nuclear magnetic resonance spectroscopy; NC, nucleocapsid protein; SAR, structure-activity relationship; UV, ultraviolet.
